# Factors Associated With the Ability To Keep Up With Technology Developments: Findings From a National Multigenerational Cross-Sectional Survey in Sweden

**DOI:** 10.2196/77930

**Published:** 2025-11-14

**Authors:** Jens Offerman, Sofi Fristedt, Steven M Schmidt, Susanne Iwarsson

**Affiliations:** 1 Department of Health Sciences, Faculty of Medicine, Lund University, Solvegatan 19, Lund, Sweden, +46 722474006; 2 School of Health and Welfare, Jonkoping University, Jonkoping, Sweden

**Keywords:** digital inclusion, aging, technology adoption, digital literacy, generational differences, health equity, ICT attitudes, information and communication technology

## Abstract

**Background:**

Digital technologies are increasingly central to supporting autonomy, health, and social participation in later life. However, disparities persist in the ability to keep up with technological developments, affecting individuals’ opportunities to benefit from digital health and social innovations.

**Objective:**

This study aimed to investigate factors associated with individuals’ self-reported ability to keep up with technological developments, focusing on generational differences, attitudes toward digital tools, and sociodemographic characteristics.

**Methods:**

We conducted a national cross-sectional online survey in Sweden with 2121 respondents aged 30 to 39 years, 50 to 59 years, and 70 to 79 years. Logistic regression analyses were used to identify associations between self-reported ability to keep up with technology and independent variables, including attitudes toward information and communication technology, gender, education, self-rated economic situation, and general health.

**Results:**

Most respondents reported being able to keep up with technological developments. Compared to the oldest generation (70-79 years), participants aged 30 to 39 years had 188% higher odds (odds ratio [OR] 2.88, 95% CI 1.84-4.53) of reporting they kept up with technology developments, and women had lower odds than men (OR 0.52, 95% CI 0.39-0.70). Positive attitudes toward information and communication technology being user-friendly (OR 1.81, 95% CI 1.21-2.73), timesaving (OR 2.03, 95% CI 1.44-2.87), and increasing independence (OR 1.99, 95% CI 1.33-2.96) were also significantly associated with keeping up.

**Conclusions:**

These findings suggest that digital inclusion in aging societies is shaped by complex and intersecting factors that go beyond age. Promoting equitable digital engagement requires addressing attitudinal, economic, and gender-related barriers and fostering inclusive technology design and support systems for both current and future generations of older adults.

## Introduction

Over the last decade, research on aging and technology has expanded extensively, and potential benefits of technology to support active and healthy aging have been identified [1-3]. Current and future generations of older adults are all immersed in the process of adopting, choosing, and using various technologies available today. The role of technology in promoting active and healthy aging is becoming increasingly relevant for people of all ages [4,5]. However, research on aging and technology has typically targeted one generation at a time, neglecting to consider the similarities and differences among and across generations. For instance, findings from a meta-analysis suggest that chronological age in itself only predicts lower technology acceptance in cases where perceived usefulness is unclear, such as with social media [6]. Instead, older adults—such as other age groups—are more likely to adopt technologies when they perceive them as valuable and relevant to their lives [7]. To facilitate technology adoption among forthcoming generations of older adults, more knowledge is imperative to nurture the development of effective services and interventions.

The decision to adopt technology is influenced by multiple factors, including perceived value, ease of use, affordability, confidence, and individual interests [7-9]. The Unified Theory of Acceptance and Use of Technology (UTAUT) provides a framework for understanding these factors, highlighting key determinants such as performance expectancy, effort expectancy, social influence, and facilitating conditions [10]. According to UTAUT, people are more likely to adopt technology when they perceive it as beneficial, easy to use, socially supported, and have access to the necessary resources and skills. This model can be particularly insightful in understanding how different generations approach technology adoption, as it considers both intrinsic factors (eg, personal motivation) and extrinsic factors (eg, social influences and accessibility) [10].

Interest in technology is not necessarily age-specific but rather depends on individual previous experiences and interests [11]. Evidence indicates that both younger and older adults report difficulties with information and communication technologies (ICTs) necessary for everyday activities [12,13]. However, older adults often report lower confidence and greater concerns about complexity, cost, and privacy, which can slow adoption [14,15]. These mixed findings point to the importance of going beyond chronological age as a factor determining technology adoption.

Still, keeping up with technology can become a challenge as we age [1]. Research has shown that people who take an early interest in technology are more likely to continue using and keeping up with technology later in life [16]. While younger generations are more inclined to be early adopters, it is important to recognize that older generations are increasingly using technology [17]. According to Lee et al [18], age disparities likely diminish over time, implying that attitudes toward and use of technology will become more similar across generations. Cross-national studies further highlight how adoption patterns vary by context. In countries with strong digital infrastructures, older adults report higher levels of technology use, whereas in other settings, barriers linked to affordability and access remain more pronounced [19,20]. Even in high-income countries such as Sweden, where overall internet use is high, 1 in 5 adults report need of assistance with digital tasks [21]. Noteworthy, this need is not limited to older adults; younger age groups are increasingly reporting difficulties with everyday digital tasks [21].

In a previous study, Offerman et al [22] identified similarities and differences between generations in their attitudes toward technology and the types of technology they wanted for active and healthy aging. In another study, Offerman et al [23] found similarities regarding what types of ICT products were used more often during the early phase of the COVID-19 pandemic. Across 3 age groups, most respondents in both studies described having the necessary technological knowledge to meet their everyday needs and to keep up with technology developments. Such findings suggest that the digital divide is not necessarily between younger and older generations, but rather between groups of users with different characteristics and their interest and ability to keep up with technology. Understanding the underlying factors associated with the ability to keep up with technology can help refine technological design. This, in turn, enables the development of products and services that better suit the diverse needs of people of all ages and ensure that everyone can keep up with technological advancements.

Accordingly, there is a need to study factors that influence the adoption and usage of digital technology, such as people’s self-perceptions of keeping up with technological developments, and to identify how these perceptions are associated with attitudes toward technology and sociodemographic characteristics. Taken together, this suggests that technology adoption is shaped not only by age but also by confidence, motivation, and contextual circumstances. However, most prior studies have examined one generation at a time, providing limited insight into similarities and differences across cohorts. Existing evidence is contradictory because some studies predict that generational gaps in technology adoption will diminish over time [18], whereas others suggest that persistent disparities in resources, confidence, and motivation will sustain inequality [19,24]. It remains underexplored how individuals across different generations perceive their ability to keep up with technological developments and which sociodemographic and attitudinal factors are associated with these perceptions.

In this study, *keeping up with technology developments* refers to individuals’ self-reported ability to follow and adapt to ongoing technological changes in everyday life. The phenomenon at target captures aspects of perceived competence and confidence, as well as willingness to engage with new technologies, rather than objectively measured skills or behaviors. This construct is conceptually linked to digital competence and perceived digital inclusion as described in previous research [19]. Addressing this phenomenon allows us to better understand multigenerational patterns of technology adoption and inform inclusive strategies for active and healthy aging.

In this study, the term digital technology refers to ICT (eg, computers, smartphones, internet services, and applications) and other digital tools that support health and everyday activities. This study builds on an earlier study from the same survey [22], which examined generational attitudes toward technology in a broad sense. While that work mapped overall attitudes, less is known about the specific factors associated with people’s self-reported ability to keep up with ongoing technological developments. Accordingly, the purpose of this study was defined based on the findings from the previous study from the GenerationTech project. Focusing on sociodemographic characteristics and attitudinal factors, this study builds on the UTAUT framework [10] and prior research showing that these variables are central for technology adoption. The purpose was to investigate factors associated with the self-reported ability to keep up with technology developments among people from 3 age groups representing different generations, taking attitudes toward technology and sociodemographics into consideration. The analyses were guided by the following research question: Which sociodemographic (age, gender, education, health, and economic resources) and attitudinal factors (perceived usefulness, ease of use, time-saving potential, and independence support of ICT and digital household devices) are associated with the self-reported ability to keep up with technological developments?

## Methods

### Respondents, Sampling, and Recruitment

Operationalizing generational categories as 3 distinct age groups to avoid overlap, a random sample was drawn from the Swedish State Personal Address Register, representing men and women stratified in 3 age cohorts (30‐39, 50‐59, and 70‐79 years).

Kantar Sifo (KS; a company with documented experience from large-scale data collection) acquired 10,000 addresses from Swedish State Personal Address Register in August 2019. Different numbers of addresses were included for each age cohort to compensate for the fact that younger people tend to have lower response rates based on KS’s data collection experience. The survey was conducted between August and October 2019.

KS used a comprehensive strategy to achieve the highest possible response rates. Postal letters containing informed consent information, survey web links, and unique login details to an online questionnaire were sent to potential respondents. Nonresponders were followed up with a postal reminder after 1 week, and if they still did not respond, they were contacted up to 8 times by trained staff via telephone. During these calls, respondents were reminded about the survey and given the alternatives of completing it via a telephone interview or receiving a postal version.

These efforts resulted in a final sample of 2121 respondents (n=1081 men, 51%; and n=1040 women, 49%). The response rate was 22% for men and 21% for women across 3 age groups (for details, see a study by Offerman et al [22]). Most of the 2121 respondents were born in Sweden and had at least completed compulsory school education. The youngest generation had a higher education level than the older generations and reported better general health, whereas the oldest generation rated their general health and economy lower than the other generations. Table 1 provides detailed sample characteristics.

**Table 1.** Characteristics of survey sample (n=2121).

Characteristics	Age 30‐39 y (n=639), n (%)	Age 50‐59 y (n=703), n (%)	Age 70‐79 y (n=779), n (%)
Sex			
Men	316 (49)	345 (49)	420 (54)
Women	323 (51)	358 (51)	359 (46)
Education			
Compulsory school	14 (2)	30 (4)	217 (28)
High school	153 (24)	244 (35)	111 (14)
Polytechnic	85 (14)	83 (12)	130 (17)
University	383 (60)	341 (49)	314 (41)
Self-rated economy for technology needs			
Good	333 (52)	382 (55)	305 (40)
Fairly good	228 (36)	239 (34)	338 (44)
Fairly bad	55 (9)	51 (7)	76 (10)
Bad	21 (3)	25 (4)	52 (6)
Self-rated general health^a^			
Excellent	130 (21)	120 (17)	64 (8)
Very good	264 (41)	275 (39)	242 (32)
Good	182 (29)	213 (31)	302 (39)
Fair	52 (8)	69 (10)	143 (19)
Poor	9 (1)	21 (3)	17 (2)
Self-rated life satisfaction^a^			
Excellent	96 (15)	115 (17)	120 (16)
Very good	290 (46)	307 (44)	301 (39)
Good	183 (29)	193 (28)	258 (34)
Fair	53 (8)	66 (9)	80 (10)
Poor	12 (2)	13 (2)	6 (1)

a Assessed using a self-reported measure based on the SF-36 Health Survey [25].

In this study, the term “generation” was used pragmatically to refer to 3 age groups: 30 to 39 years, 50 to 59 years, and 70 to 79 years. These groups were chosen to reflect meaningful differences in historical exposure to digital technologies and societal transitions while avoiding overlapping of adjacent cohorts. That is, the age groups represent distinct life-course positions rather than fixed generational identities, capturing variation in digital familiarity, health needs, and societal expectations regarding aging. Generational categories, defined by birth years, assume shared experiences that shape attitudes and behaviors. These categories highlight broad patterns, such as how historical and cultural contexts influence technology adoption. For example, generational cohorts may share exposure to specific technologies or societal shifts that have shaped their comfort and confidence with technologies.

### Data Collection

Data were collected through a structured questionnaire developed for the GenerationTech project [22], based on qualitative findings [11] involving the same age groups as this study and relevant scientific literature, focusing on technology use, attitudes, and adoption. The survey was constructed to align with the research objectives and included questions grounded in theories of technology adoption and digital inclusion [10,26]. The survey included 24 questions about attitudes toward and adoption of technology, for example, “I have no problem keeping up with technology developments.” Questions addressed experiences and attitudes toward ICT and household devices, for instance, if the respondent perceives these save time, are useful, support independence, and are user-friendly. The survey also included 7 questions about respondent characteristics, such as education, occupation, housing, civil state, and country of birth, as well as self-rated general health, life satisfaction, and self-rated economy to cover technology needs (Multimedia Appendix 1). To ensure data quality and usability, a pilot study, followed by minor revisions to the questions, was conducted before full-scale data collection. It took approximately 10 to 15 minutes to complete the survey.

KS performed regular quality control of data during data collection, focusing on correct, complete, and logical recording in the database. Researchers monitored the process to identify potential errors, and KS communicated with the research team when needed. For more details, see the Method section of the study by Offerman et al [22].

### Data Analyses

Descriptive statistics were used for sample demographics. Binary logistic regression was implemented to analyze the factors associated with the respondents’ reported ability to keep up with technological developments. The dependent variable was whether respondents reported keeping up with technology developments. This question had 4 response alternatives, ranging from strongly disagree to strongly agree. To facilitate the data analysis because of the nonnormal distribution and skewness of the data, the response alternatives were merged and dichotomized into agreeing or not agreeing (yes or no). In addition, this approach was chosen to enhance the interpretability for a broad audience, facilitate consistency across related studies, and strengthen the comparability of outcomes. Independent variables included gender, belonging to a specific generation (ie, age group), attitudes toward ICT and digital household devices (eg, perceieved usefulness, user-friendly, timesaving, and a means to increase independence), and sociodemographic characteristics, such as education, self-rated health, self-rated economy for technology, and life satisfaction. To identify potential multicollinearity, a correlation matrix was established. None of the independent variables met the criterion for exclusion (cutoff value >0.7). Logistic regressions were first computed univariably, followed by a multivariable model, including all independent variables. Cases with missing values on the dependent variable were excluded. For independent variables, respondents with incomplete data were excluded from the regression models where it was deemed relevant. As the proportion of missing data was low, no imputation was performed. Data were not normally distributed but met the assumptions of the statistical tests used. SPSS Statistics 29 (IBM) was used for the data analyses. The alpha level was set to *P<*.05.

### Ethical Considerations

This study was based on a quantitative, cross-sectional survey conducted as part of the GenerationTech project. The Swedish Ethical Review Authority (No: 2019‐02072) approved the study. KS performed the sampling, recruitment, and data collection on behalf of and in collaboration with the research team (for details, see a study by Offerman et al [22]). All respondents gave their informed consent.

## Results

A majority of the respondents reported having the ability to keep up with technology developments. In the univariable models, compared to the oldest generation, the odds of reporting having no problems keeping up with technology developments were 308% higher among the youngest generation and 72% higher in the middle-aged generation. Attitudes toward ICT all showed a significant positive association with the dependent variable. Having a university education compared to compulsory school-level education was associated with 187% higher odds of reporting having no problems keeping up with technology developments (Table 2).

**Table 2.** Univariable and multivariable logistic regression models of factors associated with the reported ability to keep up with technology developments (n=2121). Note: The dependent variable is reported ability to keep up with technology developments. Independent variables are generation, gender, self-rated life satisfaction, self-rated health, self-rated economy to cover technology needs, attitudes toward ICT^a^, and household devices. Model fit (Nagelkerke *R*²)=0.218. Numbers are rounded to nearest integer.

Factor	Univariable model		Multivariable model	
	OR^b^ (95% CI)	*P* value	OR (95% CI)	*P* value
Generation				
30‐39 y	4.08 (2.71-6.12)	<.001	2.88 (1.84-4.53)	<.001
50‐59 y	1.72 (1.27-2.33)	<.001	1.36 (0.95-1.93)	.09
70‐79 y	Ref^c^	Ref	Ref	Ref
Gender				
Male	Ref	Ref	Ref	Ref
Female	0.55 (0.42-0.73)	<.001	0.52 (0.39-0.70)	<.001
Attitudes toward ICT				
Perceieved usefulness (ref no)	1.79 (1.36-2.35)	<.001	1.33 (0.96-1.84)	.08
User friendly (ref no)	2.96 (2.09-4.19)	<.001	1.81 (1.21-2.73)	.004
Timesaving (ref no)	2.72 (2.05-3.61)	<.001	2.03 (1.44-2.87)	<.001
Make me independent (ref no)	2.37 (1.69-3.32)	<.001	1.99 (1.33-2.96)	<.001
Attitudes toward household devices				
Perceieved usefulness (ref no)	1.15 (0.87-1.51)	.32	0.75 (0.53-1.05)	.09
User friendly (ref no)	1.31 (0.99-1.73)	.06	0.84 (0.59-1.18)	.32
Timesaving (ref no)	1.36 (1.03-1.80)	.03	0.89 (0.63-1.27)	.51
Make me independent (ref no)	0.94 (0.69-1.28)	.69	0.68 (0.46-0.99)	.04
Education level				
Compulsory school	Ref	Ref	Ref	Ref
High school	2.34 (1.56-3.52)	<.001	1.32 (0.82-2.12)	.25
Polytechnic	2.19 (1.38-3.49)	<.001	1.55 (0.94-2.58)	.09
University	2.87 (1.99-4.13)	<.001	1.45 (0.95-2.21)	.09
General health				
Excellent	1.95 (1.21-3.15)	.006	1.31 (0.74-2.30)	.35
Very good	1.83 (1.30-2.57)	<.001	1.47 (0.99-2.17)	.05
Good	Ref	Ref	Ref	Ref
Bad	0.63 (0.44-0.90)	.01	0.85 (0.56-1.29)	.44
Life satisfaction				
Excellent	1.73 (1.09-2.74)	.02	1.19 (0.69-2.04)	.52
Very good	1.47 (1.07-2.03)	.02	1.06 (0.73-1.53)	.77
Good	Ref	Ref	Ref	Ref
Bad	0.73 (0.49-1.11)	.14	0.89 (0.55-1.44)	.65
My economy covers my technology need				
Good	1.08 (0.79-1.47)	.62	0.71 (0.50-0.99)	.04
Fairly good	Ref	Ref	Ref	Ref
Bad	0.47 (0.33-0.68)	<.001	0.55 (0.36-0.83)	.004

a ICT: information and communication technology.

b OR: odds ratio.

c Ref: reference.

In the multivariable model, compared to the oldest generation, the odds in the youngest generation were 188% higher of reporting having no problems keeping up with technology developments. Women were less likely (odds ratio [OR] 0.52, 95% CI 0.39-0.70) to report themselves as keeping up with technology developments compared to men. In the multivariable model, attitudes such as user-friendly (OR 1.81, 95% CI 1.21-2.73), time-saving (OR 2.03, 95% CI 1.44-2.87), and technology as a means to increase independence (OR 1.99, 95% CI 1.33-2.96) all showed significant associations with the dependent variable, reporting having no problems to keep up with technology developments. Education showed no significant associations in the multivariable model. Respondents who rated their economy as good (OR 0.71, 95% CI 0.50-0.99) or bad (OR 0.55, 95% CI 0.36, 0.83) compared to those who rated their economy fairly good were less likely to report keeping up with technology developments (Table 2). The final multivariable model showed a Nagelkerke R² of 0.218.

## Discussion

### Principal Findings

Our investigation of factors associated with the reported ability to keep up with technology among current and future generations of older adults reveals essential insights into the dynamics of technology adoption and its perceived benefits. Both the youngest and the middle-aged generations were more likely than the oldest generation to report having the ability to keep up with technology developments, with a majority across all generations indicating the same. This study highlights key factors associated with whether individuals report keeping up with technology, for instance, attitude toward technology, self-rated economy for technology needs, age, and gender.

Generational differences in technology adoption deserve attention. Younger individuals, particularly those aged 30 to 39 years, are more likely to perceive themselves as technologically up to date compared to older adults, such as those aged 70 to 79 years. UTAUT is a useful framework to explore how generational differences in constructs, such as social influence, effort expectancy, and facilitating conditions, may explain these disparities in technology adoption [26]. In addition, these differences could stem from varying levels of exposure to technology, particularly through education and professional experiences and environments, which allow younger individuals to integrate technology more seamlessly into their lives [27]. In contrast, older generations often require more time and support to adapt to new technologies, facing unique challenges due to limited prior exposure [15,16,26]. However, Lee et al [18] suggested that these age-related disparities in technology adoption may diminish over time, as attitudes and usage patterns across generations gradually converge. In line with this, in a recent study [23], we found that respondents across 3 generations reported having the necessary technological knowledge to meet their everyday needs, although this competence was more pronounced in the youngest generation. Investigating this further, in this study, most respondents reported keeping up with technological developments. Taken together, these findings suggest that age-related disparities in technology adoption may be influenced not only by exposure but also by individual perceptions of the benefits and usability of technology. According to UTAUT, people are more likely to adopt technology when they perceive it as beneficial to their daily lives [26]. This perceived benefit, related to performance expectancy, likely contributes to the reported ability to keep up with technological developments. Thus, the digital divide may not solely be between younger and older adults, but between user groups with varying levels of perceived usefulness and expected performance outcomes.

The results show that people with a positive attitude toward ICT are more likely to report keeping up with technology developments. Previous research has shown that older adults are more likely to adopt new technology when they perceive it as easy to use and beneficial for their independence and daily life [16,28]. Similarly, our study shows that individuals who view technology as user-friendly, time-saving, and a means to increase independence are more inclined to report having the ability to keep up with technology developments. These attitudes are crucial motivators for learning and engaging with technology [1,29] and critical determinants of technology adoption [10]. UTAUT helps explain this by suggesting that as facilitating conditions (eg, access to technology and support) improve, older adults may experience fewer barriers to adopting and using technology [26].

The proactive aging research approach, introduced by Iwarsson et al [30], aligns with these findings by recognizing the dynamic interactions between individuals and their environments throughout the aging process. This approach considers aspects throughout the life course, addressing both current and future generations of older adults, from earlier stages of aging into later life. It focuses on the potential and challenges associated with aging, including cognitive, psychological, physical, and social factors, all of which can influence technology adoption among older adults. Promoting positive attitudes toward technology through campaigns and programs that highlight its benefits can support goals toward proactive aging [30] and encourage broader acceptance and engagement with technology among older adults [31,32].

Surprisingly and somewhat difficult to interpret, both those who responded that their economy covers their technology needs as good or bad are less likely to report keeping up with technology compared to those who report their economy as fair. This could indicate that individuals at economic extremes may either feel less motivation to engage with technology due to financial security and make other priorities in life, or they lack the resources and motivation due to financial constraints. However, previous literature indicates that individuals with higher socioeconomic status tend to have better access to technology [33]. Our finding adds nuance, showing that access might not be enough to elicit feelings of being able to keep up with the rapid technological developments. Our findings suggest that individuals with financial security may be slower to adopt new technologies, whereas those with limited means often struggle to access them. This gap can significantly deepen existing disparities, as financial stability can postpone technology adoption, thereby widening the technological divide and further marginalizing those who are less fortunate in an increasingly digital landscape. It is also possible that participants with financial security adopt alternative solutions or invest their resources in other valued areas (eg, travel, sports, or social activities), making digital engagement less of a priority. In this sense, digital exclusion may stem not only from economic constraints but also from economic choice, reinforcing that motivation, interest, and perceived usefulness are central to understanding technology adoption across different groups. However, we would like to emphasize that our interpretation of this unexpected finding remains cautious and should be further explored in future research.

Gender differences play a significant role in technology engagement, with women less likely than men to report keeping up with technological developments. Similarly, a cross-national meta-analysis found that men consistently rate their technology abilities higher than women, although this difference is narrowing over time [34]. This disparity may stem from differences in access to technology, confidence levels, and societal norms that influence technology use and career choices [35]. In fact, men tend to overestimate their technological abilities, whereas women underestimate theirs [36]. Research suggests this is partly due to exclusive design processes and inadequate testing involving women [37]. Addressing these disparities is crucial to reduce the digital divide between genders. Enhancing women’s confidence in technology requires more inclusive design processes and equitable testing across genders. Ensuring that digital technologies are designed inclusively and tested equitably can improve accessibility, usability, and overall engagement, ultimately reducing gender disparities in technology adoption [37].

Moreover, while higher education was influential in univariate analyses, more than doubling the odds of reporting keeping up with technology developments, it did not remain significant in the multivariable model. Individuals with higher education levels are more likely to report keeping up with technology compared to those with only compulsory education. This suggests that education equips individuals with critical thinking skills and technical knowledge necessary for engaging with new technologies [27,38]. Higher education fosters critical thinking abilities, which are crucial for understanding and adapting to new technologies. This educational advantage translates into greater ease in adopting and using new technologies, reinforcing the perception of keeping up with technological developments [38]. Again, there are notable differences among subgroups of the aging population deserving explicit attention regarding the prerequisites for technology adoption.

### Implications

Addressing generational diversity and socioeconomic disparities in technology adoption should be a priority in digitalization policies. Our findings highlight the importance of fostering positive attitudes toward technology, ensuring equitable access, and addressing disparities in perceived usefulness and competence. Targeted strategies are essential to support people in keeping up with technology along the life course and as they age, particularly those from older generations [31].

Support systems tailored to meet individual needs within different generations, due to socioeconomic backgrounds, are essential to prevent further digital exclusion [8]. Examples include digital literacy programs embedded in community services, intergenerational mentoring initiatives, and locally available “digital helpdesks” where individuals can receive hands-on support. Policies should be informed by individuals’ actual needs, recognizing that both financial constraints and financial security can influence technology engagement. Providing economic support and incentives for technology purchases and training can help individuals with lower economic means access and adopt new technologies [8]. Such support can ensure that financial barriers do not hinder technological engagement, fostering a more inclusive digital society [32]. Furthermore, inclusive design and testing processes must be implemented to reduce gender disparities and enhance usability for all [37]. This means involving users from diverse age groups and socioeconomic backgrounds in co-design processes and ensuring that interfaces are intuitive, adaptable, and responsive to different skill levels. This, in turn, could help reduce the intimidation factor associated with new technology [39]. Policies and interventions should go beyond access to promote confidence, motivation, and meaningful digital participation, with sustained investment in digital skills development across the life course [19]. Without such targeted strategies and interventions, there is a risk that digital transformation will reinforce existing inequalities, leaving certain groups behind as they struggle to keep up with technological developments.

### Strengths and Limitations

It should be kept in mind that the cross-sectional design restricts the ability to disentangle age, cohort, and period effects. Generational categories were used to describe different sociotechnical contexts, and the observed differences are best understood as cross-sectional associations rather than causal generational effects. Longitudinal research is required to clarify these dynamics.

The strength of this study lies in the nationwide sample, which was randomly selected from the Swedish population. However, despite extensive efforts to use a broad sampling frame and issue multiple reminders, the response rate was low, particularly among younger generations. Nevertheless, the sample reflects the demographic composition of the Swedish population. For instance, 78% of respondents had at least a high school education, compared to 85% in the general population. A substantial majority of respondents (90%) were born in Sweden, and approximately 75% resided in larger cities. The diversity in socioeconomic backgrounds and nationalities among respondents contributed to a heterogeneous survey sample. Still, it is also possible that individuals who are less digitally engaged or less confident in technology use were underrepresented, which may have led to an overestimation of the perceived ability to keep up with technology and an underestimation of the challenges faced by the least digitally included groups. Therefore, while the findings remain generally relevant, they should be interpreted with consideration of these factors and potential bias.

Another inherent limitation is that participation in a survey on digital technology may have favored individuals with greater interest or competence in digital technology, potentially leading to overestimation of engagement. Nonresponse bias may also have occurred, with less confident users underrepresented. As with all self-reported data, recall and social desirability bias are possible. Although survey weights were applied to enhance representativeness, some subgroups may still be over- or under-represented.

It would have been beneficial to include questions about the respondents’ actual use and previous experience with technology. However, designing the survey required balancing comprehensiveness with an acceptable respondent burden. In retrospect, adding these questions would have made it easier to draw conclusions about the respondents’ level of interest, knowledge, and competence. It would also have made it possible to study whether there is an association between using and keeping up with technology, which is an interesting question for future research.

The dichotomization of the outcome variable is another limitation deserving comment because it entailed a loss of nuance and may have obscured subtle variations in perceptions. Despite these trade-offs, we ultimately deemed this the most appropriate analytical strategy. In addition to enhancing communicative clarity, dichotomization supported consistency across studies and facilitated comparability of outcomes, thereby strengthening the coherence of the overall analysis.

### Conclusions

This study underscores the multifaceted factors associated with the ability to keep up with technology among current and future generations of older adults. Applying a proactive aging approach to research on aging and technology highlights the value of adopting a generational perspective to understand similarities and distinct challenges faced by different age groups. Across generations, perceived ease of use and benefits such as enhanced independence and time-saving capabilities are essential motivators for technology adoption. Addressing educational, economic, gender, and attitudinal barriers, fostering positive attitudes toward technology, promoting user-friendly designs, and encouraging inclusivity across demographic divides are critical steps toward bridging the digital divide and fostering a more inclusive environment for technological engagement.

The results underscore the importance of encouraging individuals to prepare for technological and environmental changes to enhance their quality of life as they age. By implementing targeted strategies that consider the differences between generations, society can ensure that all individuals have the opportunity to keep up with technology and benefit from its potential to promote active and healthy aging for current as well as future generations of older adults. This includes policies that support lifelong digital skill development, interventions such as intergenerational mentoring and community-based digital support, and design practices that prioritize accessibility and co-creation with diverse users. Taken together, these efforts can help translate research insights into concrete actions that promote equitable digital inclusion across the life course.

## Supplementary material

10.2196/77930 Multimedia Appendix 1 Questionnaire used for the survey study.

## Abbreviations

ICT: information and communication technology
KS: Kantar Sifo
OR: odds ratio
UTAUT: Unified Theory of Acceptance and Use of Technology
