# Supplementary material for: Factors Associated With the Ability To Keep Up With Technology Developments: Findings From a National Multigenerational Cross-Sectional Survey in Sweden
Source: JMIR Aging. 2025 Nov 14;8:e77930. doi: 10.2196/77930 (PMC12617988; doi:10.2196/77930)
Supplement: Multimedia Appendix 1 [file aging-v8-e77930-s001.docx]

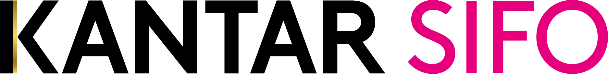


**GenerationTech survey**

This survey includes questions on technical products (i.e. objects, but also services based on technology) in general and technical products for active and healthy ageing in particular. What constitutes active and healthy ageing is personal to the individual, and is linked to being able to do what you want and think is important, for example. There are no right or wrong answers. We are interested in your opinion on these issues, regardless of how old you are. We would like to investigate whether there are any differences and similarities between the use of technology and attitudes of different generations (people aged 30-39, 50-59 and 70-79) to new technology. Based on the responses given by you and other participants, we are aiming to develop new intelligence on how technology can be used and designed to support activity and health as people age.

**How to fill in the form.**

***Here is an example:***

**To what extent do you agree with the statement “*It is sunny today”***

| Strongly disagree | Agree to an extent | Agree to a great extent | Strongly agree |
| --- | --- | --- | --- |
| **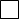** | **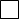** | **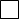** | **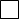** |
| **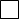**  🗶 | **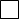** | **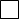** | **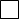** |
| **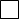** | **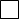** | **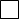** | **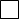**  🗶  🗶 |

If you think the statement is

incorrect, put a cross in the

box on the far left as follows:

If you think the statement is

correct, put a cross like this:

If you accidentally put a cross in the wrong box, correct it by crossing out the whole box. Then put a cross in the correct box as follows:

The form will be read optically by a computer.

🗶

🗶

So please keep the crosses inside the boxes if you can. Put a cross like this: Not like this:

Preferably use a ballpoint pen, and not ink or pencil.

1. **What type or types of technical products would you like to use yourself to support active and healthy ageing?** *Select all options that you consider relevant.*

| ☐ | Household appliances (e.g. dishwasher, washing machine, microwave) |
| --- | --- |
| ☐ | Home entertainment devices (e.g. TV, radio, video, game consoles) |
| ☐ | Technical assistive devices (e.g. walkers, wheelchairs) |
| ☐ | Activity sensors (e.g. for physical activity) |
| ☐ | Equipment for physical activity (e.g. gym equipment, walking poles or bicycle) |
| ☐ | Personal health sensors (e.g. for measuring blood pressure, blood sugar) |
| ☐ | Technical medical products (e.g. pacemakers, insulin pumps) |
| ☐ | Personal security alarms |
| ☐ | “Smart homes” |
| ☐ | Sensors for healthcare and care services in the home (e.g. night cameras, fall sensors) |
| ☐ | Robots to assist with various activities in the home |
| ☐ | Robots that can interact socially with humans |
| ☐ | Motor vehicles |
| ☐ | Social media |
| ☐ | Internet-based shopping |
| ☐ | Internet-based public services (e.g. digital contact with healthcare and care services, libraries, public authorities) |
| ☐ | Other, what? …………………...…………………...…………………...…………… |

1. **For what reasons would you personally like to use technical products to support active and healthy ageing?** *Select all options that you consider relevant.To:*

| ☐ | Save time |
| --- | --- |
| ☐ | Look after myself |
| ☐ | Create security |
| ☐ | Keep in touch with family and friends |
| ☐ | For fun and entertainment |
| ☐ | Map health |
| ☐ | Raise the alarm in case of a fall or illness |
| ☐ | Control objects in my home environment |
| ☐ | Get service |
| ☐ | Make purchases |
| ☐ | Exercise |
| ☐ | Technology cannot promote active and healthy ageing |
| ☐ | Other reason, what? …………………...…………………...…………………...……… |

1. **What are your attitudes on the opportunities for people in general to support active and healthy ageing using technical products?**

| ☐ | New technical products need to be developed to make this possible |
| --- | --- |
| ☐ | Technical products are available for this, but availability is limited by society |
| ☐ | Technical products are available for this, but not everyone can afford them |
| ☐ | Technical products are available and are already available to everyone today |

1. **What kind of design do you prefer for technical products that are intended to support your own (future) active and healthy ageing?**

| ☐ | That they have the same design as technical products that I used earlier in life |
| --- | --- |
| ☐ | That they have the same design that resembles something I used before |
| ☐ | That they are designed specifically for older people |
| ☐ | I prefer the very latest technical products |
| ☐ | Other design, please specify:……………...…………………...…………………...… |
|  |  |

1. **Who do you think should be involved in** **the development of** **technical products to be used to support active and healthy ageing?**  *Select all options that you consider relevant.*

| ☐ | Designers |
| --- | --- |
| ☐ | Companies that produce technology |
| ☐ | Resellers in the sector |
| ☐ | Representatives of healthcare and care services |
| ☐ | Private individuals who will be using the products |
| ☐ | Researchers |
| ☐ | Other, please specify: ……………...…………………...…………………...…………. |
|  |  |

1. **Who do you think should be involved in choosing technical products that can be used to support active and healthy ageing?**
   *Select all options that you consider relevant.*

| ☐ | The individual themselves |
| --- | --- |
| ☐ | The individual’s relatives |
| ☐ | Healthcare and nursing professionals |
| ☐ | Companies that produce technology |
| ☐ | Resellers in the sector |
| ☐ | Other, please specify: ……………...…………………...…………………...………….. |
|  |  |

1. **How do you think personal technical assistive devices (e.g. walkers, wheelchairs) or welfare technology (e.g. security alarms, night cameras) to support active and healthy ageing should be paid for?**

| ☐ | Society should pay |
| --- | --- |
| ☐ | The individual should pay |
| ☐ | Relatives should pay |
| ☐ | The community and the individual should share the cost |
| ☐ | Other, state how: ……………...………………...…………………...…………….. |

## **Significance of technical products over time, technology shifts**

Here are some questions about how important you think technical products have been and are in your day-to-day life. As before, the term refers to technical products and objects, but also to services based on technology

1. **New technical products are constantly evolving, but which technology shift had the** **greatest impact on your day-to-day life: when you were a child (until you were about 18 years old)?**
   *When I/my family got access to:*

| ☐ | Analogue home phone |
| --- | --- |
| ☐ | Washing machine at home |
| ☐ | Radio |
| ☐ | Car |
| ☐ | Black and white TV |
| ☐ | Colour TV |
| ☐ | Home computer |
| ☐ | Mobile phone |
| ☐ | Dial-up internet |
| ☐ | Game consoles |
| ☐ | Broadband |
| ☐ | Smartphone |
| ☐ | Other, please specify: ……………...………………...…………………...…………….. |

1. **New technical products are constantly evolving, but which technology shift had the greatest impact on your day-to-day life: as an adult (from about the age of 19 until today)?**

When I got access to:

| ☐ | Analogue home phone |
| --- | --- |
| ☐ | Washing machine at home |
| ☐ | Radio |
| ☐ | Car |
| ☐ | Black and white TV |
| ☐ | Colour TV |
| ☐ | Home computer |
| ☐ | Mobile phone |
| ☐ | Dial-up internet |
| ☐ | Game consoles |
| ☐ | Broadband |
| ☐ | Smart mobile phone |
| ☐ | Other, please specify: ……………...………………...…………………...…………….. |

## **Digital services**

Here are some questions about digital services.

1. **How satisfied or dissatisfied are you with your ability to influence whether you want to use digital services for public services and banking?**

| ☐ | Very satisfied |
| --- | --- |
| ☐ | Satisfied |
| ☐ | Neither satisfied nor dissatisfied |
| ☐ | Dissatisfied |
| ☐ | Very dissatisfied |

1. **If you indicated dissatisfied/very dissatisfied above, why are you dissatisfied?**

| ☐ | Don’t want to use, but have no choice |
| --- | --- |
| ☐ | Want to use more often, but no service is available |
| ☐ | Other, please specify: ……………...………………...…………………...……………. |

1. **How would you prefer to access public services, banking, etc. if you could choose?**

| ☐ | Via digital services |
| --- | --- |
| ☐ | Via a combination of digital services and personal service |
| ☐ | Via personal service |

1. **What is your main reason for this choice?**

| ☐ | Accessibility |
| --- | --- |
| ☐ | Convenience |
| ☐ | Saving time |
| ☐ | Protecting my privacy |
| ☐ | Personal safety |
| ☐ | Want to talk to a person |
| ☐ | Other reason: ……………………………………………………………………… |

1. **In general, how do you prefer to pay for goods and services?**

| ☐ | Cash |
| --- | --- |
| ☐ | By debit or credit card |
| ☐ | Via app (e.g. Swish, parking apps) |
| ☐ | Internet banking |
| ☐ | Other: ..…………...……………...…………………...………………… |

1. **What is your main reason for this choice?**

| ☐ | Accessibility |
| --- | --- |
| ☐ | Convenience |
| ☐ | Saving time |
| ☐ | Protecting my privacy |
| ☐ | Personal safety |
| ☐ | Other reason: ……………………………………………………………………… |

1. **Who do you think should be responsible for ensuring that you, as a private individual, have the necessary knowledge to use digital services from companies, public authorities, banks, etc.?**

*Select all options that you consider relevant.*

| ☐ | It is my own responsibility |
| --- | --- |
| ☐ | The owner or provider of the service (e.g. public authorities, companies) |
| ☐ | Course organisers (e.g. study associations) |
| ☐ | Associations and organisations (e.g. trade unions, senior citizens’ organisations) |
| ☐ | The local authority (e.g. via the library) |
| ☐ | Other, please specify: ……………...……………...…………………...……………… |

1. **In practice,** **who ensures that you, as a private individual, have the necessary knowledge to use digital services from companies, public authorities, banks, etc.?**

*Select all options that you consider relevant.*

| ☐ | It is my own responsibility |
| --- | --- |
| ☐ | The owner or provider of the service (e.g. public authorities, companies) |
| ☐ | Course organisers (e.g. study associations) |
| ☐ | Associations and organisations (e.g. trade unions, senior citizens’ organisations) |
| ☐ | The local authority (e.g. via the library) |
| ☐ | Other, please specify: ……………...……………...…………………...……………… |

## **Advice on technical products**

1. **Who do you turn to for purchasing advice in the first instance when you are planning to buy a new technical product?** *Select one option.*

| ☐ | Relatives |
| --- | --- |
| ☐ | Friends |
| ☐ | The Web |
| ☐ | Social media |
| ☐ | News media |
| ☐ | Trade press |
| ☐ | Consumer information |
| ☐ | Product tests |
| ☐ | Price information |
| ☐ | Resellers |
| ☐ | Other, please specify: ……………...…………….…………………...………………… |
| ☐ | None, I sort it out for myself without any of the above |

1. **If I have problems with my own technical product, do I usually resolve them?**

*Select all options that you consider relevant.*

| ☐ | Myself (e.g. trying things out) |
| --- | --- |
| ☐ | With the help of relatives |
| ☐ | With the help of friends |
| ☐ | With the help of telephone support |
| ☐ | With the help of information on the Internet |
| ☐ | With the help of the company where I bought it |
| ☐ | Via municipal services (e.g. the library) |
| ☐ | Other, please specify: ……………...……………....……………...………………… |

## **Experiences with technical products**

Here are some questions about your opinion on different types of technical products (objects and services) that we ask you to consider.

1. **I think in general that household appliances (washing machine, kitchen appliances, etc.):**

*Select all options that you consider relevant.*

| ☐ | Are useful |
| --- | --- |
| ☐ | Are user-friendly |
| ☐ | Meet necessary needs |
| ☐ | Are practical |
| ☐ | Are intrusive |
| ☐ | Save time |
| ☐ | Are reliable |
| ☐ | Are safe to use |
| ☐ | Give me independence |
| ☐ | Limit my privacy |
| ☐ | None of the above |

1. **I think in general that information and communication technology (computers, smartphones, etc.):**
   *Select all options that you consider relevant.*

| ☐ | Are useful |
| --- | --- |
| ☐ | Are user-friendly |
| ☐ | Meet necessary needs |
| ☐ | Are practical |
| ☐ | Are intrusive |
| ☐ | Save time |
| ☐ | Are reliable |
| ☐ | Are safe to use |
| ☐ | Give me independence |
| ☐ | Limit my privacy |
| ☐ | Am forced to use them |
| ☐ | None of the above |

1. **To what extent do you agree with the following statements about technical products (goods and services)?**

|  | Strongly disagree | Agree to an extent | Agree to a great extent | Strongly agree |
| --- | --- | --- | --- | --- |
| Technical products are usually designed to be easy to use regardless of the user’s experience and ability. | ☐ | ☐ | ☐ | ☐ |
| Technical products are usually designed so that it is easy to understand how to use them | ☐ | ☐ | ☐ | ☐ |
| Technical products are usually designed so that it is easy to understand how to use them. | ☐ | ☐ | ☐ | ☐ |
| The technical products of today are of better quality than those developed in the past. | ☐ | ☐ | ☐ | ☐ |
| Technical products developed today have a shorter service life than older technologies. | ☐ | ☐ | ☐ | ☐ |
| Technical products are developed because it is technically possible to do so, rather than working on the basis of what people need. | ☐ | ☐ | ☐ | ☐ |
| Technical products are developed for profit, rather than to meet people’s needs | ☐ | ☐ | ☐ | ☐ |

1. **What is your opinion on the following statements about the choice, use, etc. of technical products?**

|  | Strongly disagree | Agree to an extent | Agree to a great extent | Strongly agree |
| --- | --- | --- | --- | --- |
| When it comes to technical products, I always want the latest/newest models | ☐ | ☐ | ☐ | ☐ |
| Price is important when I am choosing technical products. | ☐ | ☐ | ☐ | ☐ |
| I prefer technical products that can be used flexibly for a variety of purposes/functions | ☐ | ☐ | ☐ | ☐ |
| I prefer technical products (e.g. cars, washing machines and dishwashers) that have standard features rather than extras | ☐ | ☐ | ☐ | ☐ |
| I prefer technical products that can be connected and controlled from a device, e.g. a mobile phone. | ☐ | ☐ | ☐ | ☐ |
| I prefer technical products that can be connected and controlled from a device, e.g. a mobile phone. | ☐ | ☐ | ☐ | ☐ |
| I prefer technical products similar to the ones I have had in the past | ☐ | ☐ | ☐ | ☐ |
| I have no problem keeping up with technology | ☐ | ☐ | ☐ | ☐ |
| I find it easy to learn new technologies and technical products | ☐ | ☐ | ☐ | ☐ |
| Ensuring that the technical products I use are environmentally sustainable is important to me | ☐ | ☐ | ☐ | ☐ |
| I am worried by the fact that important social functions are dependent on technology. | ☐ | ☐ | ☐ | ☐ |

1. **What is your opinion on the following statements about information and communication technology and digital services?**

|  | Strongly disagree | Agree to an extent | Agree to a great extent | Strongly agree |
| --- | --- | --- | --- | --- |
| My personal data is handled securely when I use information and communication technology (e.g. computer, smartphone) for digital public services   (e.g. at the Swedish Tax Agency, Swedish Social Insurance Agency) | ☐ | ☐ | ☐ | ☐ |
| My personal data is handled securely when I use information and communication technology (e.g. computer, smartphone) to manage my finances and make purchases. | ☐ | ☐ | ☐ | ☐ |
| My personal data is handled securely when I use information and communication technology (e.g. computer, smartphone) on social media | ☐ | ☐ | ☐ | ☐ |
| I generally trust the information I can find on the Internet | ☐ | ☐ | ☐ | ☐ |
| I like to use social media to keep in touch with my friends | ☐ | ☐ | ☐ | ☐ |
| I like to use social media to get news and social information | ☐ | ☐ | ☐ | ☐ |

## **Questions about you**

1. **Which of the following describes you best?**

| ☐ | Single |
| --- | --- |
| ☐ | Married or cohabiting |
| ☐ | Living apart together |
| ☐ | Widow or widower |
| ☐ | None of the above |

1. **Number of people in my household who are…

   a)** under 18 years old: ………………………………... persons

   **b)** Aged 18 and older (including yourself): ..…….. persons
2. **Where were you born?**

| ☐ | Sweden |
| --- | --- |
| ☐ | Another country: ……………...……………...…………………...……………… |

1. **If born in a country other than Sweden: How long have you lived in Sweden?** ………...………… years
2. **What is the highest level of education you have completed?**

| ☐ | Primary school, elementary school, secondary school or similar |
| --- | --- |
| ☐ | Upper secondary education |
| ☐ | Vocational school, vocational college or similar |
| ☐ | University or college education |

1. **My main occupation at the moment is:**

| ☐ | Studies |
| --- | --- |
| ☐ | Professional |
| ☐ | On parental leave |
| ☐ | Pensioner |
| ☐ | Unemployed |
| ☐ | Other: ……………...……………...…………….……………...………………………. |

1. **How well do you think your finances cover your/your family’s technology needs at the moment?**

| ☐ | Well |
| --- | --- |
| ☐ | Fairly well |
| ☐ | Fairly poorly |
| ☐ | Poorly |

1. **In general, would you say that…**

|  | Excellent | Very good | Good | Fair | Poor |
| --- | --- | --- | --- | --- | --- |
| **…your health is?** | ☐ | ☐ | ☐ | ☐ | ☐ |
| **…your satisfaction with life is?** | ☐ | ☐ | ☐ | ☐ | ☐ |
